# Atypical teratoid rhabdoid tumor in the cavernous sinus of a toddler presenting with oculomotor nerve palsy

**DOI:** 10.1007/s00381-014-2407-6

**Published:** 2014-03-30

**Authors:** Nami Inoue, Hiroyoshi Watanabe, Kazumi Okamura, Mika Sakaki, Teruyoshi Kageji, Shinji Nagahiro, Shoji Kagami

**Affiliations:** 1 Department of Pediatrics, The University of Tokushima Graduate School, Tokushima, Japan; 2 Department of Pathology, The University of Tokushima Graduate School, Tokushima, Japan; 3 Department of Neurosurgery, The University of Tokushima Graduate School, Tokushima, Japan

**Keywords:** Atypical teratoid rhabdoid tumor, Cavernous sinus, Oculomotor nerve palsy, Childhood

## Abstract

**Introduction:**

Atypical teratoid rhabdoid tumor (ATRT) is a rare, highly malignant, and aggressive tumor of infancy. Although the prognosis of ATRT has been extremely poor, recently, the first prospective study for ATRT demonstrated improvement of prognosis. On the other hands, oculomotor nerve palsy is rare in children and the most frequent etiology is congenital. To our knowledge, only a few ATRT cases presenting with oculomotor nerve palsy have been reported, but ATRT originating from the cavernous sinus (CS) has not yet been reported.

**Case report:**

An 18-month-old girl with right oculomotor nerve palsy was admitted, and a small mass in the right CS was detected with brain MRI. Although she received steroid pulse therapy and antimicrobial therapy, the mass continued to enlarge. One month after admission, the mass was partially resected and diagnosed as ATRT. Multimodal therapy including anthracycline-based chemotherapy, intrathecal therapy, and cranial irradiation was performed. Twenty-nine months after resection, she was alive without tumor relapse, but the oculomotor nerve palsy persisted.

**Conclusion:**

This is the first reported case of ATRT located in the CS presenting with oculomotor nerve palsy. This case was successfully treated with partial removal of the tumor, a new chemotherapy regimen for ATRT and cranial X-ray irradiation.

## Introduction

Atypical teratoid rhabdoid tumor (ATRT) is a rare, highly malignant, and aggressive tumor of infancy. ATRT was classified as an embryonal grade IV neoplasm by the WHO in 1993 [1]. Although ATRT can originate from anywhere in the central nervous system (CNS), tumor location is distributed equally in the infratentorial and supratentorial regions, and other regions are infrequent [2, 3]. Symptoms depend on tumor location. To date, there has been no established standard treatment for ATRT, and the prognosis has been extremely poor [2–6]. However, recently, the first prospective study for ATRT demonstrated improvement of prognosis [7].

Oculomotor nerve palsy is rare in children and the most frequent etiology is congenital [8, 9]. To our knowledge, only a few ATRT cases presenting with oculomotor nerve palsy have been reported [10, 11], but ATRT originating from the cavernous sinus (CS) has not yet been reported. Described is a case of ATRT in the CS that presented with oculomotor nerve palsy and was successfully treated with multimodal therapy.

## Case

**Fig. 1:**
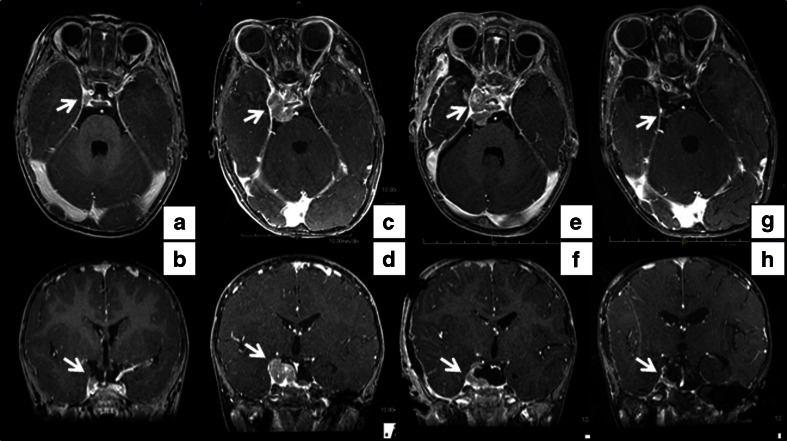
Brain MRI with gadolinium enhancement (axial and coronal sections). On admission day 1, MRI revealed a 9 × 4 mm tumor at the right cavernous sinus (**a**, **b**). Three weeks after admission, the cavernous sinus tumor had rapidly increased to 30 × 20 mm in size. The tumor invaded the cavernous sinus to the pontine cistern and compressed a part of the pons (**c**, **d**). About 50% of the tumor was resected by surgery (**e**, **f**). Six weeks after starting chemotherapy, the tumor size had reduced, and only a 7 × 4 mm tumor remained at the pontine cistern (**g**, **h**)

**Fig. 2:**
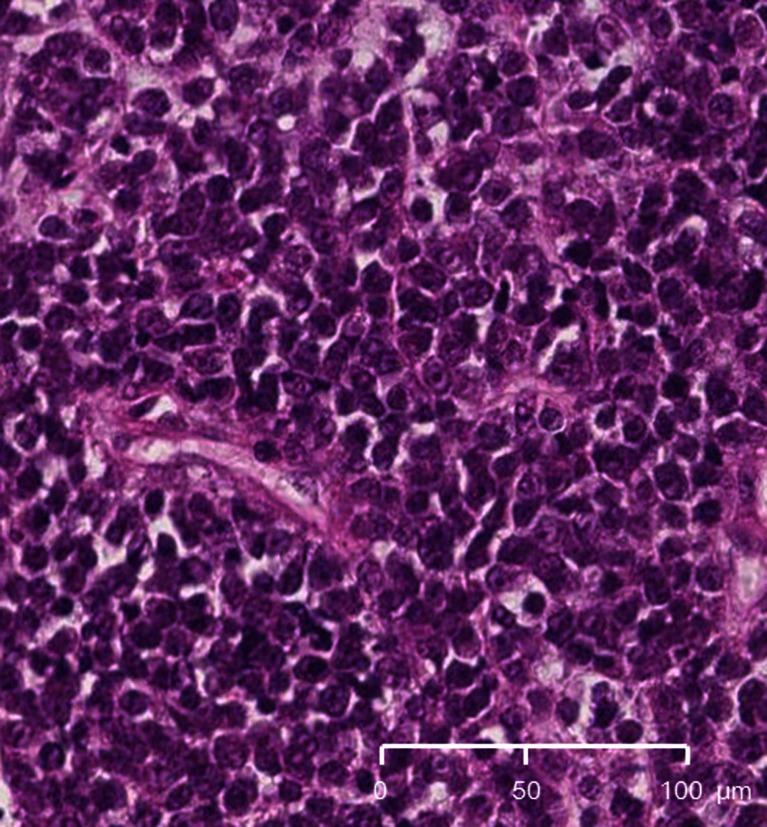
Pathological examination of the resected specimen. Hematoxylin and eosin staining reveal diffuse proliferation of small undifferentiated cells

An 18-month-old girl presented with right ptosis of 7-day duration. She was born prematurely at 31 weeks and 5 days of gestational age, because of premature rupture of membranes, and was delivered by cesarean section due to breech presentation. Her body weight at birth was 1,624 g. There was no evidence of asphyxia or traumatic episodes around delivery. Her growth and development were not problematic. At the age of 18 months, her parents noticed sudden onset right ptosis. She suffered neither infectious disease nor cranial trauma preceding the appearance of ptosis. Although her general condition had not changed, she was admitted to our hospital because her ptosis had progressed over 7 days. On admission, she was afebrile and her vital signs were normal. Physical and neurological examination did not reveal abnormal signs, except for right ptosis. Eye examination was performed by an ophthalmologist. The right eyelid was droopy, and the right pupil was dilated and non-reactive to light. There was exotropia and hypotropia of the right eye position. The symptoms did not resolve with Tensilon. Although the results of blood, urine, and cerebrospinal fluid (CSF) examinations were unremarkable, brain magnetic resonance imaging (MRI) revealed a 9 × 4 mm tumor at the right CS (Fig. 1a, b). There was no evidence of a vascular lesion there with magnetic resonance angiography (MRA). The etiology of her right oculomotor nerve palsy was considered to be a CS tumor. However, neurosurgical intervention (i.e., a biopsy or a resection of the tumor) was considered too difficult. The differential diagnoses included inflammatory disease (including Tolosa-Hunt syndrome) and lymphoma, and therefore, systemic corticosteroid pulse therapy was initiated on admission day 7, but the nerve palsy did not improve with appropriate medical treatment. Next, antimicrobial therapy was selected with suspicion of bacterial or fungal infection. Administration of meropenem, voriconazole, and liposomal amphotericin B showed no efficacy, and serological and culture examinations demonstrated no evidence of infectious disease. Three weeks after admission, she developed vomiting, and brain MRI revealed that the CS tumor was rapidly enlarging. It had grown to 30 × 20 mm with invasion into the pontine cistern with compression of a part of the pons (Fig. 1c, d). On admission day 31, the neurosurgery team performed a right frontotemporal craniotomy and partial tumor resection. According to postoperative MRI, the extent of tumor resection was about 50% (Fig. 1e, f). Pathologically, the tumor consisted of diffuse proliferation of small undifferentiated cells which resembled those of medulloblastoma (Fig. 2). Immunohistochemically, a small number of the cells were positive for SMA, EMA, and CD99. They were negative for CAM5.2, AE1/AE3, synaptophysin, GFAP, neurofilament, CD20, CD3, CD45RO, CD79a, and TdT. Lack of nuclear reactivity for INI-1 was recognized. Thus, the tumor was diagnosed as ATRT. Although chest and abdominal enhanced computed tomography did not detect another lesion, CSF examination 10 days after surgery revealed tumor cell proliferation. She was diagnosed with right CS ATRT with CSF dissemination.

Seven weeks after admission, chemoradiotherapy was initiated according to the Intergroup Rhabdomyosarcoma Study III protocol [7]. At that time the tumor size returned to the same size as before resection. This protocol consisted of anthracycline-based chemotherapy, intrathecal therapy, and cranial irradiation. Brain MRI, 6 weeks after starting chemotherapy, showed that the tumor size was dramatically reduced, and a 7 × 4 mm lesion remained at the pontine cistern (Fig. 1g, h). Six weeks after starting chemotherapy, malignant cells in the CSF were undetectable. Along with chemotherapy, she received X-ray irradiation against the primary tumor bed with a total dose of 54 Gy in 1.8 Gy fractions using intensity-modified delivery. During 13 months of chemoradiotherapy, serious and uncontrollable toxicity did not occur. At present, 29 months after initial resection, brain MRI revealed only a 5 × 5 mm residual lesion without enhancement at the pontine cistern, which has not changed in size since completion of chemoradiotherapy. However, she continues to have right oculomotor nerve palsy and disturbance of speech development.

## Discussion

In children, oculomotor nerve palsy is uncommon. Excepting congenital cases, the causes of oculomotor nerve palsy are trauma, neoplasm, vascular abnormalities, and inflammation in the region anywhere the nerve passes (such as the midbrain, cavernous sinus, orbital fissure, orbit, and subarachnoid space) [8, 9]. In the present case, the tumor within the CS was considered to have caused the oculomotor nerve palsy. The described etiologies for CS tumors have included infectious disease, Tolosa-Hunt syndrome (THS), aneurysm, thrombosis, and neoplasms [12]. Although neoplasms originating from the CS are rare in children, several case reports concerning malignant lymphoma in the CS have been published [13, 14]. In the present case, MRA ruled out vascular lesions. Although tumor biopsy is necessary for diagnosis, in general, neurosurgical biopsies of CS tumors are employed as a last resort. So, corticosteroids were first administered for diagnostic treatment of THS. It should be noted that steroid therapy can be effective for neoplasms such as lymphoma [14]. The present case did not respond to steroid therapy, so THS was excluded. Both bacterial and fungal infections are part of the differential diagnosis of CS tumors, but the present case had no evidence of infection. Finally, partial resection of the tumor and a pathological examination was performed, and ATRT was diagnosed.

ATRT is a rare but extremely aggressive malignant tumor of infancy. ATRT can occur at any site within the CNS, and the symptoms are associated with the location of the tumor. Although cranial nerve palsy has been described as a symptom of ATRT, the majority of cases of abducens or facial nerve palsy are due to infratentorial tumors [2]. To our knowledge, only two case reports of ATRT with oculomotor nerve palsy have been published. One case was a 6-week-old boy with ATRT located in the interpeduncular cistern [10], and the other was a 56-year-old woman with ATRT located in the sella [11]. The present patient had a novel ATRT with regards to the location in the CS. Although neoplasms in the CS are rare in children, and neurosurgical biopsy of a CS tumor is technically difficult, rapidly increasing tumors should be suspected to be aggressive malignant tumors such as ATRT, and therefore, prompt biopsy is necessary.

As previously reported, the prognosis of ATRT has been extremely poor [2–6]. Tekautz et al. reported that the 2-year event-free and overall survival rates of children younger than 3 years of age were 11 and 17%, worse than in older patients [15]. In a previous study, ATRT patients received chemotherapy designed for other pediatric malignant brain tumors (such as medulloblastomas and primitive neuroectodermal tumors), and many ATRT patients younger than 3 years have had cranial X-ray irradiation refused or delayed in order to avoid long-term neurocognitive complications [4–6, 15]. The role of high-dose chemotherapy with hematopoietic stem cell rescue for ATRT is still controversial [16]. However, the first prospective clinical trial for newly diagnosed ATRT in children published in 2009 [7] demonstrated significant improvement of prognosis, with a 2-year progression-free survival rate of 53% and an overall survival rate of 70%. In that trial, the treatment regimen after resection consisted of anthracycline-based chemotherapy, intrathecal therapy, and cranial X-ray irradiation regardless of age. This chemoradiotherapy regimen was adopted in the present report, and the patient has survived without relapse for 29 months after tumor resection.

A recent retrospective study indicated that radiotherapy improves survival outcome for ATRT [17], but there is no published review for long-term complications of ATRT survivors. In compensation for prolonged survival, the present patient may experience long-term complications, including neurocognitive impairment due to cranial X-ray irradiation. Bernstein et al. reported the first cohort of ATRT patients treated with proton beam radiotherapy in 2013 [18]. They described that 9 of 10 patients were alive without evidence of disease with a median follow-up of 27.3 months. Proton beam therapy is expected to reduce damage to normal tissue. Although the assessment of long-term complications, especially neurocognitive outcomes, after proton beam radiation compared with X-ray irradiation may require long-term follow-up, the results of their study encourage to the adaptation of proton therapy as a treatment strategy for ATRT patients, particularly for younger patients. It is expected that the original strategy for ATRT patients, such as high-dose chemotherapy with hematopoietic stem cell rescue, proton beam radiotherapy, and molecular targeting therapy, which is equal in efficacy and has decreased long-term sequelae compared with cranial X-ray irradiation, will be established in the future.

## References

[1] Kleihues P, Burger PC, Scheithauer BW (1993). Histological typing of tumours of central nervous system. World Health Organization International Histological Classification of Tumours.

[2] Rorke LB, Packer RJ, Biegel JA (1996). Central nervous system atypical teratoid/rhabdoid tumors of infancy and childhood: Definition of an entity. J Neurosurg.

[3] Lafay-Cousin L, Hawkins C, Carret AS (2012). Central nervous system atypical teratoid rhabdoid tumours: the Canadian Paediatric Brain Tumour Consortium experience. Eur J Cancer.

[4] Packer RJ, Biegel JA, Blaney S (2002). Atypical teratoid/rhabdoid tumor of the central nervous system: Report on workshop. J Pediatr Hematol Oncol.

[5] Geyer JR, Sposto R, Jennings M (2005). Multiagent chemotherapy and deferred radiotherapy in infants with malignant brain tumors: a report from the Children's Cancer Group. J Clin Oncol.

[6] Von Hoff K, Hinkes B, Dannenmann Stern E (2011). Frequency, risk-factors and survival of children with atypical teratoid rhabdoid tumors (AT/RT) of the CNS diagnosed between 1988 and 2004, and registered to the German HIT database. Pediatr Blood Cancer.

[7] Chi SN, Zimmerman MA, Yao X (2009). Intensive multimodality treatment for children with newly diagnosed CNS atypical teratoid rhabdoid tumor. J Clin Oncol.

[8] Schumacher-Feero LA, Yoo KW, Solari FM, Biglan AW (1999). Third cranial nerve palsy in children. Am J Ophthalmol.

[9] Holmes JM, Mutyala S, Maus TL, Grill R, Hodge DO, Gray DT (1999). Pediatric third, fourth, and sixth nerve palsies: a population-based study. Am J Ophthalmol.

[10] Wykoff CC, Lam BL, Brathwaite CD (2008). Atypical teratoid/rhabdoid tumor arising from the third cranial nerve. J Neuroophthalmol.

[11] Arita K, Sugiyama K, Sano T, Oka H (2008). Atypical teratoid/rhabdoid tumour in sella turcica in an adult. Acta Neurochir.

[12] Kline LB, Hoyt WF (2001). The Tolosa-Hunt syndrome. J Neurol Neurosurg Psychiatry.

[13] Choi HK, Cheon JE, Kim IO (2008). Central skull base lymphoma in children: MR and CT features. Pediatr Radiol.

[14] Demirkaya M, Sevinir B, Ozdemir O, Nazlioglu HO, Okan M (2010). Lymphoma of the cavernous sinus mimicking Tolosa-Hunt syndrome in a child. Pediatr Neurol.

[15] Tekautz TM, Fuller CE, Blaney S (2005). Atypical teratoid/rhabdoid tumors (ATRT): Improved survival in children 3 years of age and older with radiation therapy and high-dose alkylator-based chemotherapy. J Clin Oncol.

[16] Garrè ML, Tekautz T (2010). Role of high-dose chemotherapy (HDCT) in treatment of atypical teratoid/rhabdoid tumors (AT/RTs). Pediatr Blood Cancer.

[17] Buscariollo DL, Park HS, Roberts KB, Yu JB (2012). Survival outcomes in atypical teratoid rhabdoid tumor for patients undergoing radiotherapy in a Surveillance, Epidemiology, and End Results analysis. Cancer.

[18] De Amorim BK, Sethi R, Trofimov A (2013). Early clinical outcomes using proton radiation for children with central nervous system atypical teratoid rhabdoid tumors. Int J Radiation Oncol Biol Phvs.

